# Supplementary figures and images for: Acute heat priming promotes short-term climate resilience of early life stages in a model sea anemone
Source: PeerJ. 2023 Dec 5;11:e16574. doi: 10.7717/peerj.16574 (PMC10704996; doi:10.7717/peerj.16574)

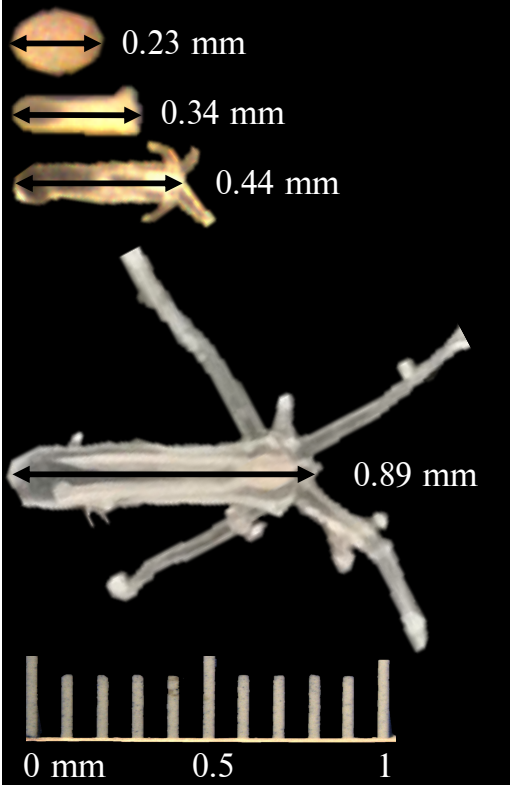

Supplement: Supplemental Information 1 — Cropped images of Nematostella vectensis larvae and juveniles at various lengths and stages of development, and a cropped image of a ruler used to determine the body column lengths. Images were collected using identical microscope settings, then the ruler image was used to set scale and a line was drawn along the major axis of the body column of each animal (black lines with arrowheads) to determine the length. White text displays the values resulting from the example measurements. [file peerj-11-16574-s001.pdf]

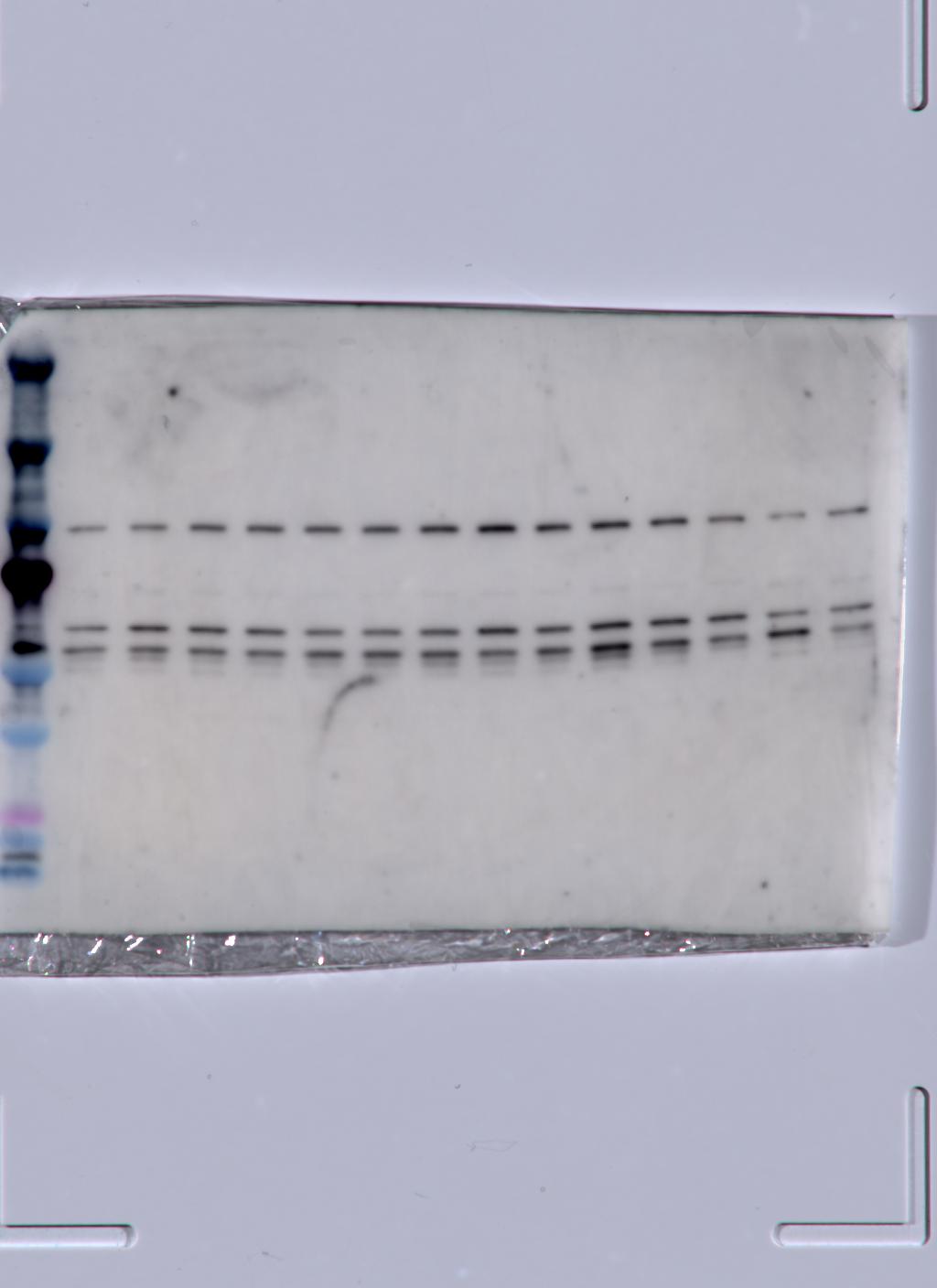

Supplement: Supplemental Information 9 [file peerj-11-16574-s009.jpg]
